# Correspondence

**Published:** 1903-09

**Authors:** E. J. Mellish

**Affiliations:** El Paso, Texas


### Correspondence.

EL PASO, TEXAS, August 9, 1903.

*Dr. F. E. Daniel, Editor Texas Medical Journal, Austin, Texas.*

DEAR DOCTOR: Owing to the press of other duties, I have only just now finished the perusal of the July "Red-Back." I merely want to say "amen" to two of the articles in that issue, which appeal to me especially, namely: the one on appendicitis, by Dr. Matthews, of Austin, and the one entitled "Experience vs. Theory," by Dr. Loggins, of Willis. There is much food for thought in each of them. I am glad to see that the treatment (palliative, of course, to prepare for operation later) of certain cases of acute appendicitis by the method exploited by A. J. Oschner is coming more and more into vogue. This method is applicable to practically all the cases of appendicitis which come to the surgeon too late for the early operation and too early for the late operation. A glance at the latest statistics of these two surgeons should be sufficient to convince the most skeptical that Dr. Oschner's claims are based upon something more substantial than wind. It is begging the question to claim that Dr. Deaver's series of cases represent very much graver conditions on the average than the series reported by Dr. Oschner and the series reported by Mayo, of Minnesota. One has only to read the report of Deaver's cases to become convinced that several of the fatal cases in the series could probably have been saved by means of less radical measures, such as would have been carried out by Oschner or any of his discerning disciples.

"Experience vs. Theory." I do not know the writer of that little article, but a thoughtful man must have written it. It is quite the fashion for the younger men in our profession to think that the older ones are away behind in the fight with disease. As a matter of fact, a large amount of experience is absolutely essential to the attainment of the best results in the practice of any branch of medicine. A good stock of "horse sense" and experience combined, with good preliminary training and knowledge, a fair acquaintance with the advances of the times, and, above all, a conscientious regard for the welfare of each patient treated, will go farther toward saving of life than all the theory on two continents.

How common is such obstetric "bosh," as that quoted by Dr. Loggins. It is enough to make the angels weep to observe the way in which some eminent specialists handle obstetric cases. They seem to think only of pathology or of some particular organ, forgetting the fact that there is a person around that organ or concerned in that pathology.

Yours very truly,

E. J. MELLISH.

